# Supplementary material for: Rapid response of fly populations to gene dosage across development and generations
Source: Nat Commun. 2024 May 29;15:4551. doi: 10.1038/s41467-024-48960-4 (PMC11137061; doi:10.1038/s41467-024-48960-4)
Supplement: Supplementary file 3 — Description of Additional Supplementary Files [file 41467_2024_48960_MOESM3_ESM.pdf]

Description of Additional Supplementary Files

File Name: Supplementary Data 1
Description: Variants with recurrent changes across multiple populations between Generation 3 and 7.

File Name: Supplementary Data 2
Description: Marker genes of yolk cluster that are differentially expressed in the evolved line.

File Name: Supplementary Data 3
Description: Differentially expressed genes between the evolved line and the other two samples.

File Name: Supplementary Data 4
Description: Map of eGFP-Bicoid construct used to generate the 4x*bcd* line in this study.

File Name: Supplementary Movie 1
Description: Light-sheet imaging of eGFP-tagged Bicoid throughout embryonic development of 4x*bcd* embryos.
