# Supplementary material for: Rapid response of fly populations to gene dosage across development and generations
Source: Nat Commun. 2024 May 29;15:4551. doi: 10.1038/s41467-024-48960-4 (PMC11137061; doi:10.1038/s41467-024-48960-4)
Supplement: Supplementary file 9 — Reporting Summary [file 41467_2024_48960_MOESM9_ESM.pdf]

nature portfolio

Corresponding author(s): Xueying Li, Justin Crocker

Last updated by author(s): Apr 30, 2024

Reporting Summary

Nature Portfolio wishes to improve the reproducibility of the work that we publish. This form provides structure for consistency and transparency in reporting. For further information on Nature Portfolio policies, see our Editorial Policies and the Editorial Policy Checklist.

Statistics

For all statistical analyses, confirm that the following items are present in the figure legend, table legend, main text, or Methods section.

n/a	Confirmed	
☐	☒	The exact sample size (n) for each experimental group/condition, given as a discrete number and unit of measurement
☐	☒	A statement on whether measurements were taken from distinct samples or whether the same sample was measured repeatedly
☐	☒	The statistical test(s) used AND whether they are one- or two-sided<br>*Only common tests should be described solely by name; describe more complex techniques in the Methods section.*
☐	☒	A description of all covariates tested
☐	☒	A description of any assumptions or corrections, such as tests of normality and adjustment for multiple comparisons
☐	☒	A full description of the statistical parameters including central tendency (e.g. means) or other basic estimates (e.g. regression coefficient) AND variation (e.g. standard deviation) or associated estimates of uncertainty (e.g. confidence intervals)
☐	☒	For null hypothesis testing, the test statistic (e.g. F, t, r) with confidence intervals, effect sizes, degrees of freedom and P value noted<br>*Give P values as exact values whenever suitable.*
☒	☐	For Bayesian analysis, information on the choice of priors and Markov chain Monte Carlo settings
☒	☐	For hierarchical and complex designs, identification of the appropriate level for tests and full reporting of outcomes
☒	☐	Estimates of effect sizes (e.g. Cohen's d, Pearson's r), indicating how they were calculated

Our web collection on statistics for biologists contains articles on many of the points above.

Software and code

Policy information about availability of computer code

Data collection	Confocal images were acquired with Zen software by Zeiss. Sequencing data were collected with Illumina NextSeq 500 or 2000.
Data analysis	Images were analyzed with FIJI (ImageJ 1.54f). Custom R codes and source data are deposited at: https://git.embl.de/xuli/rapid-response-of-fly-populations-to-gene-dosage-across-development-and-generations.<br>Software versions:<br>ImageJ 1.54f<br>bowtie2 2.4.4-GCC-11.2.0<br>picard 3.1.0-Java-17<br>freebayes 1.3.6-foss-2021b-R-4.1.2<br>GATK 4.2.3.0-GCCcore-11.2.0-Java-11<br>bcftools 1.16-GCC-11.3.0<br>annovar 2020-06-08<br>cell ranger 6.0.1<br>FIMtrack 2.1<br>samtools 1.18-GCC-12.3.0<br><br>python 3.9.6-GCC-11.2.0<br>scrublet 0.2.3<br><br>R 4.2.2-foss-2022b for bioinformatic analysis<br>seurat 3.9.9.9010

harmony 0.1.1
dplyr 1.1.0
ggplot2 3.4.1
ggalluvial 0.12.5
stringr 1.5.0
cowplot 1.1.1
reshape2 1.4.4
R 4.3.2 for phenotypic analysis
ggplot2 3.4.4
reshape2 1.4.4
dplyr 1.1.4
stringr 1.5.1
scales 1.3.0
ggnewscale 0.4.9
nlme 3.1-163
emmeans 1.10.0
cowplot 1.1.3
FactoMineR 2.11
factoextra 1.0.7
corrplot 0.92

For manuscripts utilizing custom algorithms or software that are central to the research but not yet described in published literature, software must be made available to editors and reviewers. We strongly encourage code deposition in a community repository (e.g. GitHub). See the Nature Portfolio guidelines for submitting code & software for further information.

Data

Policy information about availability of data

All manuscripts must include a data availability statement. This statement should provide the following information, where applicable:

- Accession codes, unique identifiers, or web links for publicly available datasets
- A description of any restrictions on data availability
- For clinical datasets or third party data, please ensure that the statement adheres to our policy

The WGS and snRNA-seq reads were deposited at ArrayExpress (EMBL-EBI) under experiments E-MTAB-11768 [https://www.ebi.ac.uk/biostudies/arrayexpress/studies/E-MTAB-11768] and E-MTAB-12068 [https://www.ebi.ac.uk/biostudies/arrayexpress/studies/E-MTAB-12068], respectively. The metabolomics data were deposited at METASPACE [https://metaspace2020.eu/project/li_x-2024]. All data supporting the findings of this study are available within the paper and its Supplementary Information files. Source data are provided with this paper.

Research involving human participants, their data, or biological material

Policy information about studies with human participants or human data. See also policy information about sex, gender (identity/presentation), and sexual orientation and race, ethnicity and racism.

Reporting on sex and gender	*Use the terms sex (biological attribute) and gender (shaped by social and cultural circumstances) carefully in order to avoid confusing both terms. Indicate if findings apply to only one sex or gender; describe whether sex and gender were considered in study design; whether sex and/or gender was determined based on self-reporting or assigned and methods used.<br>Provide in the source data disaggregated sex and gender data, where this information has been collected, and if consent has been obtained for sharing of individual-level data; provide overall numbers in this Reporting Summary. Please state if this information has not been collected.<br>Report sex- and gender-based analyses where performed, justify reasons for lack of sex- and gender-based analysis.*
Reporting on race, ethnicity, or other socially relevant groupings	*Please specify the socially constructed or socially relevant categorization variable(s) used in your manuscript and explain why they were used. Please note that such variables should not be used as proxies for other socially constructed/relevant variables (for example, race or ethnicity should not be used as a proxy for socioeconomic status).<br>Provide clear definitions of the relevant terms used, how they were provided (by the participants/respondents, the researchers, or third parties), and the method(s) used to classify people into the different categories (e.g. self-report, census or administrative data, social media data, etc.)<br>Please provide details about how you controlled for confounding variables in your analyses.*
Population characteristics	*Describe the covariate-relevant population characteristics of the human research participants (e.g. age, genotypic information, past and current diagnosis and treatment categories). If you filled out the behavioural & social sciences study design questions and have nothing to add here, write "See above."*
Recruitment	*Describe how participants were recruited. Outline any potential self-selection bias or other biases that may be present and how these are likely to impact results.*
Ethics oversight	*Identify the organization(s) that approved the study protocol.*

Note that full information on the approval of the study protocol must also be provided in the manuscript.

Field-specific reporting

Please select the one below that is the best fit for your research. If you are not sure, read the appropriate sections before making your selection.

☒ Life sciences ☐ Behavioural & social sciences ☐ Ecological, evolutionary & environmental sciences

For a reference copy of the document with all sections, see nature.com/documents/nr-reporting-summary-flat.pdf

Life sciences study design

All studies must disclose on these points even when the disclosure is negative.

Sample size	Sample size was determined based on common practice in developmental biology. Usually at least 10 embryos were analyzed for each condition. In the case of precious samples (samples from experimental evolution), at least 3 embryos were required.
Data exclusions	No data exclusions were performed.
Replication	Three replicates were used where relevant (e.g. quantification of triglceride level). All attempts to repeat the experiments reported in this study were successful.
Randomization	Embryos were randomly sampled from slides during microscopy. Automated imaging and analysis pipelines were used to avoid human biases in selecting samples.
Blinding	Experimenters were bind to sample labels when manual measurement was required, i.e. for counting nuclei and measuring inter-nucleus distance.

Reporting for specific materials, systems and methods

We require information from authors about some types of materials, experimental systems and methods used in many studies. Here, indicate whether each material, system or method listed is relevant to your study. If you are not sure if a list item applies to your research, read the appropriate section before selecting a response.

Materials & experimental systems

n/a	Involved in the study	
☐	☒	Antibodies
☒	☐	Eukaryotic cell lines
☒	☐	Palaeontology and archaeology
☐	☒	Animals and other organisms
☒	☐	Clinical data
☒	☐	Dual use research of concern
☒	☐	Plants

Methods

n/a	Involved in the study	
☒	☐	ChIP-seq
☒	☐	Flow cytometry
☒	☐	MRI-based neuroimaging

Antibodies

Antibodies used	Eve antibody (mouse, 2B8-concentrate, 1:20) was from Developmental Studies Hybridoma Bank. Bicoid antibody (rabbit, 1:250) was a gift from Pinar Onal and Stephen Small. The following antibodies were used at 1:500: anti-biotin (PA1-26792, goat, ThermoFisher); anti-DIG (ab6212, mouse, Abcam); Anti-FITC (A889, rabbit, ThermoFisher); Alexa 555 (A31572), Alexa 488 (A21202) and Alexa 633 (A21100) (all from ThermoFisher).
Validation	All the primary antibodies have been previously validated (Tsai et al. 2017).

Animals and other research organisms

Policy information about studies involving animals; ARRIVE guidelines recommended for reporting animal research, and Sex and Gender in Research

Laboratory animals	Drosophila melanogaster (strains w1118, VK18, VK33, Ind, CantonS). D. ananassae (TSC 14024-0371.13), D. biarmipes (TSC 14023-0361.01), D. eugracilis (from the US National Drosophila Species Stock Center), D. parabipectinata (inbred derivative of strain TSC 14024-0401.02), D. pseudoobscura (TSC 14011-0121-94 USA), D. sechellia (TSC 14021-0248-25), D. yakuba (TSC 14021-0261.01) and D. virilis (w-).
Wild animals	*Provide details on animals observed in or captured in the field; report species and age where possible. Describe how animals were caught and transported and what happened to captive animals after the study (if killed, explain why and describe method; if released, say where and when) OR state that the study did not involve wild animals.*

Reporting on sex	*Indicate if findings apply to only one sex; describe whether sex was considered in study design, methods used for assigning sex. Provide data disaggregated for sex where this information has been collected in the source data as appropriate; provide overall numbers in this Reporting Summary. Please state if this information has not been collected. Report sex-based analyses where performed, justify reasons for lack of sex-based analysis.*
Field-collected samples	*For laboratory work with field-collected samples, describe all relevant parameters such as housing, maintenance, temperature, photoperiod and end-of-experiment protocol OR state that the study did not involve samples collected from the field.*
Ethics oversight	Ethical approval was not required for Drosophila research based on European regulations.

Note that full information on the approval of the study protocol must also be provided in the manuscript.

Plants

Seed stocks	*Report on the source of all seed stocks or other plant material used. If applicable, state the seed stock centre and catalogue number. If plant specimens were collected from the field, describe the collection location, date and sampling procedures.*
Novel plant genotypes	*Describe the methods by which all novel plant genotypes were produced. This includes those generated by transgenic approaches, gene editing, chemical/radiation-based mutagenesis and hybridization. For transgenic lines, describe the transformation method, the number of independent lines analyzed and the generation upon which experiments were performed. For gene-edited lines, describe the editor used, the endogenous sequence targeted for editing, the targeting guide RNA sequence (if applicable) and how the editor was applied.*
Authentication	*Describe any authentication procedures for each seed stock used or novel genotype generated. Describe any experiments used to assess the effect of a mutation and, where applicable, how potential secondary effects (e.g. second site T-DNA insertions, mosiacism, off-target gene editing) were examined.*
